# Merkel cell carcinoma in an immunocompetent male statin user^☆^^☆☆^

**DOI:** 10.1016/j.abd.2019.01.005

**Published:** 2019-10-24

**Authors:** Isaura Azevedo Fasciani, Luisa Groba Bandeira, Neusa Yuriko Sakai Valente, Maria Fernanda Vieira Cunha Camargo

**Affiliations:** a Department of Dermatology, Hospital do Servidor Público Estadual, Instituto de Assistência Médica ao Servidor Público Estadual, São Paulo, SP, Brazil; b Department of Dermatopathology, Hospital do Servidor Público Estadual, Instituto de Assistência Médica ao Servidor Público Estadual, São Paulo, SP, Brazil

*Dear Editor*,

Merkel cell carcinoma (MCC) is a neuroendocrine primary cutaneous neoplasm with aggressive behavior. It presents high risk of local recurrence, as well as involvement of lymph nodes and distant metastases, which explains its high lethality, recommending its early recognition and treatment.

A 67-year-old male patient presented to the Dermatology Outpatient Clinic with a history of systemic arterial hypertension, type 2 diabetes mellitus, and dyslipidemia, using insulin, losartan, hydrochlorothiazide, atenolol, acetylsalicylic acid, rosuvastatin, and fibrate, six months after an asymptomatic, rapidly growing, erythematous-violaceous nodule (Fig. 1) measuring 3cm had appeared on his right leg, without palpable lymphadenomegalies. Incisional biopsy of the lesion (Figure 2, Figure 3) revealed, in the histopathology, a dermal tumor with trabecular arrangement, composed of small blue cells with scarce cytoplasm and compact nuclei. Immunohistochemistry was positive for chromogranin and cytokeratin 20, signaling the diagnosis of MCC. In the outpatient return, we requested a computed tomography (CT) scan of the chest, abdomen, and pelvis, which did not show involvement of the internal organs or lymph node enlargement. In addition to the immunosuppression tests, the serologies were also all negative. The patient was then referred for oncological surgery, where sentinel lymph node screening was performed, which did not show any neoplastic involvement. Extensive excision, measuring 7.7cm × 6.8cm × 0.8cm, was performed and the histopathology demonstrated MCC with resection margins free of involvement.

**Figure 1:**
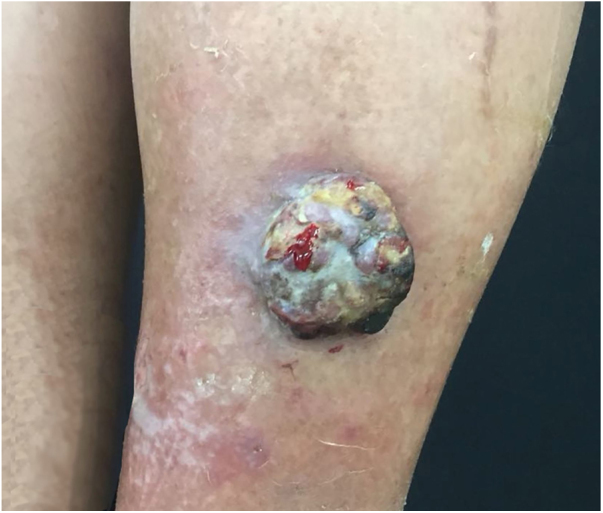
Fast-growing 3cm erythematous-violaceous nodule in right leg.

**Figure 2:**
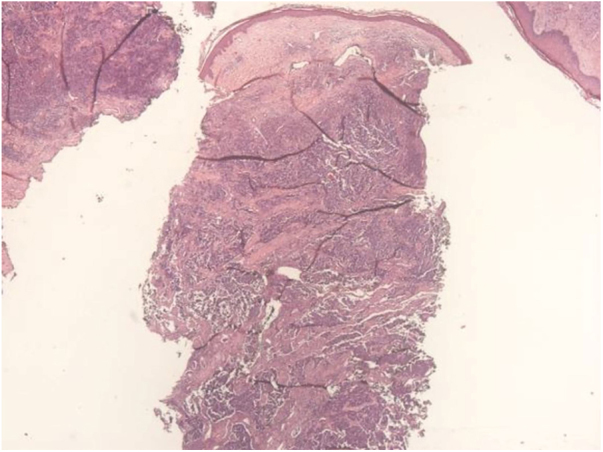
Dermal tumor with trabecular arrangement, composed of small blue cells with scarce cytoplasm and compact nuclei (Hematoxylin & eosin, x20).

**Figure 3:**
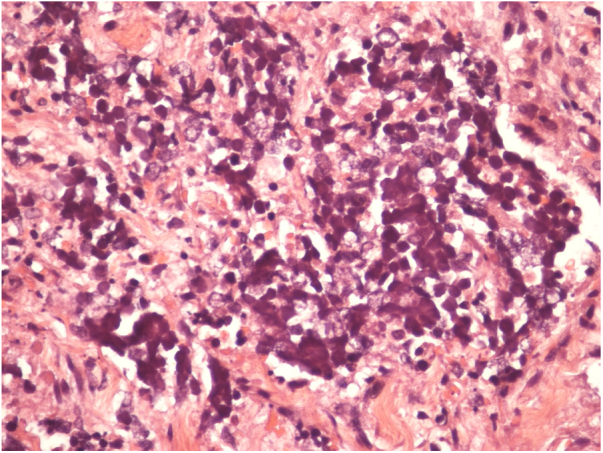
Detail of the Merkel cells (Hematoxylin & eosin, x200).

MCC is a rare, aggressive, cutaneous neuroendocrine neoplasm that has shown a significant increase in incidence over recent years, presenting as erythematous-violaceous papule or nodule, usually painless and fast growing. It appears most often in sun-exposed areas, such as the head and neck, but also occurs in the extremities, trunk, and genitalia.^1^

MCC most often affects men, with a male-to-female ratio of 1.5 to 2:1; the elderly, with a mean age of 73 years in males and 76 years in females; and the immunocompromised, such as transplant recipients and HIV carriers, in the which it usually occurs ten years earlier than the average.^2^

In 2008, MCC was related to *Polyomaviridae* infection in 80% of cases, when it was identified in tumor cells’ DNA sequences of a new human polyomavirus, known as polyomavirus of Merkel cells. The frequency of this polyomavirus ranges from 9% in individuals aged 1–4 years to 80% in individuals over 50.^3^ More recently, an increase in MCC cases has been reported in younger subjects using statins.^4^

Statins have been widely used because of their effect on lowering blood cholesterol and for having a well-established role in preventing cardiovascular and cerebrovascular events. They also has an immunomodulatory effect on the negative regulation of MHCII expression. There is a shift from Th1 (T helper cells) to Th2, which leads to the increase of B cells, activating the excessive production of antibodies, besides exerting an inhibitory effect on natural killer cells (NKs), which are crucial for natural immunity against intracellular pathogens, thus compromising immune vigilance against viral infections and predisposing to tumor cell proliferation.^5^

Hence, it is possible to infer that statins predispose to *Polyomaviridae* infection and the consequent proliferation of MCC tumor cells, a phenomenon similar as that occurring with the immunocompromised.

Because of the rarity of the tumor, there is currently no standard consensus for treatment. One recommendation is broad surgical excision with free margins of 2 cm and adjuvant or isolated radiotherapy.^5^

We report a case of MCC in an immunocompetent patient with a diagnosis below the average age of those diagnosed with MCC, with no history of other skin cancers, and a chronic statin user, thus strengthening the correlation between MCC and statin use.

## Financial support

None declared.

## Author's contributions

Isaura Azevedo Fasciani: Elaboration and writing of the manuscript.

Luisa Groba Bandeira: Obtaining, analyzing and interpreting the data; critical review of the manuscript.

Neusa Yuriko Sakai Valente: Approval of the final version of the manuscript; effective participation in research orientation; critical review of the literature; critical review of the manuscript.

Maria Fernanda Vieira Cunha Camargo: Conception and planning of the study; obtaining, analyzing and interpreting the data; critical review of the literature.

## Conflicts of interest

None declared.
